# A soft, high-density neuroelectronic array

**DOI:** 10.1038/s41528-023-00271-2

**Published:** 2023-08-22

**Authors:** Kyung Jin Seo, Mackenna Hill, Jaehyeon Ryu, Chia-Han Chiang, Iakov Rachinskiy, Yi Qiang, Dongyeol Jang, Michael Trumpis, Charles Wang, Jonathan Viventi, Hui Fang

**Affiliations:** 1 Thayer School of Engineering, Dartmouth College, Hanover, NH 03755 USA; 2 Department of Biomedical Engineering, Duke University, Durham, NC 27708 USA; 3 Department of Electrical and Computer Engineering, Northeastern University, Boston, MA 02115 USA

**Keywords:** Electrical and electronic engineering, Bionanoelectronics, Polymers, Biosensors

## Abstract

Techniques to study brain activities have evolved dramatically, yet tremendous challenges remain in acquiring high-throughput electrophysiological recordings minimally invasively. Here, we develop an integrated neuroelectronic array that is filamentary, high-density and flexible. Specifically, with a design of single-transistor multiplexing and current sensing, the total 256 neuroelectrodes achieve only a 2.3 × 0.3 mm^2^ area, unprecedentedly on a flexible substrate. A single-transistor multiplexing acquisition circuit further reduces noise from the electrodes, decreases the footprint of each pixel, and potentially increases the device’s lifetime. The filamentary neuroelectronic array also integrates with a rollable contact pad design, allowing the device to be injected through a syringe, enabling potential minimally invasive array delivery. Successful acute auditory experiments in rats validate the ability of the array to record neural signals with high tone decoding accuracy. Together, these results establish soft, high-density neuroelectronic arrays as promising devices for neuroscience research and clinical applications.

## Introduction

Neuroelectrodes are important devices for neuroscience research and neurological disorder treatments, which need to interface with the soft brain at a large spatial scale with fine resolution. Device biocompatibility is paramount to neural devices since it is needed to both prevent harm to the organism and observe neural activity in a natural, non-damaged state^1–5^. Large spatial scale is key to understanding brain function by recording from multiple brain regions with a single device^6,7^. Finally, fine spatial resolution is required for recording the basic communication system of interconnected neurons. Present devices cannot fulfill all three of these aspects simultaneously and thus need to be improved^8–11^.

Substantial progress has been made in improving the performance and biocompatibility of neural implants^12–14^. Extensive studies have demonstrated reduced damage to the brain through the use of softer materials and designs, which decrease device rigidity. Both element size and materials patterning can decrease rigidity, as shown in carbon fiber microneedle and mesh arrays^15^. Among various soft neuroelectrodes, filamentary polymer-based arrays have emerged as a promising pathway forward by combining soft, small platforms and mature lithography-based fabrication processes. These devices demonstrated improvements in chronic biocompatibility and signal stability, making them suitable for long-term neural monitoring^16^. Despite their many advances in design and performance, each electrode contact requires an individual output wire, limiting scaling from achieving more than ~100 contacts due to device size limitations.

Separately, tremendous progress has been made in conventional silicon (Si)-based neural arrays, where scaling up the number of electrodes can be achieved by utilizing active electronics, specifically complementary metal-oxide-semiconductor (CMOS) technology. For example, Neuropixels probes are high-density CMOS-based electrode arrays with 5120 recording sites distributed over four shanks and can record from 384 of those recording sites at one time. Recording otherwise from many electrode contacts simultaneously with few output wires can be achieved through active multiplexing but introduces three main issues: (1) electrical noise, (2) size constraints, and (3) poor device lifespan^8,9^. High-density and large-coverage electrodes enabled by active sensing can better detect broadband neural activity, which is known to have a high correlation with microseizures, improve the state decoder to detect the presence/absence of movement, and increase the accuracy of source localization^17–20^.

Recently, progress toward combining the biocompatibility of soft devices with the high electrode counts of actively multiplexed CMOS devices has been made. Namely utilizing ultrathin Si transistors and flexible polyimide (PI) as the substrate instead of rigid Si, demonstrating active electrodes that enable recording from multiple channels simultaneously^21–25^. These devices are designed for electrocorticographic applications at the surface of the brain and thus primarily have a sheet-like form factor with low electrode density. Additionally, active PI devices’ lifespan significantly decreases in the presence of voltage differentials between powered electronics and biological tissue^26^. When moving from polyimide to more insulative materials such as SiO_2_, device lifespan increases, but electrical noise and flexibility are sacrificed. While these issues are daunting in voltage-sensing circuit architecture, current sensing can more easily address them. Current sensing uses the traditional electrode voltage transformed through the complex impedance of the electrode^27^.

Here, we demonstrated the filamentary, actively multiplexed electrode array with 256-ch, high density and flexibility. The 256 electrodes are integrated into a small area of 2300 × 300 μm^2^, with each electrode site of only 34 × 7 μm^2^. There are several key unique features of this soft neuroelectronic array: (1) the multiplexed electrode array was fabricated in a filamentary form factor more suitable for inserting into the brain, with single-neuron-sized electrodes, all enabled by miniaturized transistor footprints; (2) the electrode multiplexing circuit utilized a 1-transistor design with current sensing, which reduced aliased noise as well as the footprint for an individual electrode pixel; and (3) the multiplexed electrode array was coupled with advanced rollable contact pads, potentially allowing device delivery via through-needle injection^28,29^. In vitro testing demonstrated electrical properties, including a high signal-to-noise ratio (SNR) of 30.1 ± 10.4 dB and great flexibility. Using a controlled motorized stand and syringe pump, the device can be precisely delivered into the target region with less than 100 μm inaccuracy. Finally, in vivo experiments validated the actively multiplexed current sensing and showed promising decoding results from the rat auditory cortex. Together, this work creates soft, high-density neuroelectronic arrays as promising devices for neuroscience studies and biomedical applications.

## Results

### Filamentary neuroelectronic array design

**Fig. 1:**
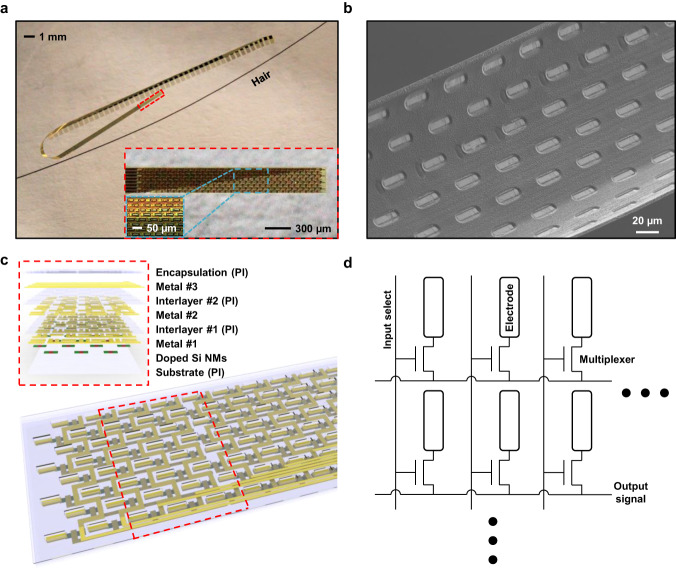
Overview of the soft, high-density neuroelectronic array. **a** Optical image of a soft, high-density neuroelectronic array with a human hair floating on the water surface. Inset: high-magnification optical images of electrode area. **b** SEM image of a device showing checkboard design and rectangular electrode sites. **c** Schematic of a device structure with an explosive view of a device, showing four different PI layers, three metal layers (Au), and a doped Si NM layer. **d** Circuit diagram of 2 × 3 array showing the sensing scheme of the device.

We designed, fabricated, and tested the soft, high-density neuroelectronic array. The width of the array was comparable to the diameter of human hair, showed great flexibility, and demonstrated lightweight by floating on the water (Fig. 1a). The device consisted of 256 electrodes (8 row inputs, 32 column outputs) uniformly distributed in an area of 2300 × 300 μm^2^ and individually multiplexed with a single Si transistor (Fig. 1a, inset). Scanning electron microscopy (SEM) image reveals the checkboard design of the electrode sites to cover a larger area with a smaller number of electrodes (Fig. 1b). The entire device had a total length of 4 cm and a width of 300 µm with micro-meshed, injectable I/O pads (Supplementary Fig. 1). Each electrode site was 34 × 7 μm^2^, similar in size to a single neuron. The rectangular shape of the electrodes achieved a smaller width of the overall device, reducing the injection footprint of the device. There were 45 total micro-meshed connection pads: 8 row-select inputs, 32 column-output signals, 3 references and 2 grounds. There were multiple reference and ground connections to enhance reliability, using the remaining pins in the 45-pin zero insertion force (ZIF) connector. The size of each meshed I/O pad was 300 μm × 5 mm with a pitch of 500 μm. The line width of the meshed pad was 10 μm with 45° diagonal traces, increasing the rollability compared to non-meshed pads.

To determine the size of the overall device and input/output (I/O) micro-meshed pads^30,31^ for the multiplexed, active electrodes, we first fabricated passive electrodes and measured their performance. The passive array was 2.7 cm long and 300 µm wide, similar to the final active electrode design (Supplementary Fig. 2a). The electrode contact size was 15 × 15 μm^2^. The device had one metal (Au) layer for electrode contacts and wiring and two PI layers for the substrate and encapsulation, yielding a total device thickness of ~2 μm. The electrode contact impedances were within an acceptable range for neural recording at 76 ± 20 kΩ^32,33^, with high uniformity (Supplementary Fig. 2b). The device was soaked in 37 °C phosphate-buffered saline (PBS) solution for 12 weeks without a noticeable change in impedance (Supplementary Fig. 2c).

The soft device incorporated a nanomembrane Si transistor array with a 3-metal (Au) layer design using thin PI as the substrate, interlayer dielectrics, and encapsulation, achieving a total thickness of only ~4.5 μm (Fig. 1c). Specifically, a bottom PI layer served as the substrate, with the two PI interlayers separating the metal layers, while another top PI layer acted as the encapsulation to define the electrodes and protect the interconnects. The first metal layer was responsible for neural sensing, transistor channel construction and row-select input wires. The next two metal layers were used for column outputs. We divided column-output wires into two metal layers to fit narrow interconnects between electrodes, ultimately reducing the device width. The sample 3 × 2 array presents sensing scheme and connection of the device (Fig. 1d). Each electrode is connected with a transistor, which acts as a multiplexer. The gate of the transistor is the input selection. The signal is recorded from the drain of the transistor and outputs the signal through source. All gates in the same column share a single output wire, and all sources in the same row also share a single wire, significantly reducing the number of output wires compared to conventional passive electrode arrays.

Our soft neuroelectronic array incorporated on-site, active multiplexing. In this work, we utilized a current-sensing strategy rather than traditional voltage sensing to (1) decrease aliased noise, (2) reduce the footprint of each pixel, and (3) potentially increase the device lifetime. In our previous actively multiplexed neuroelectrode devices, the electrode unit used a 2-transistor design with voltage sensing at each electrode (the ‘pixel’) (Supplementary Fig. 3a). In the 2-transistor architecture, neural voltage signals were sensed through the gate of a buffer transistor (T1) and then sampled in time using a multiplexing transistor (T2). The multiplexed output signal was connected to a remote recording system for filtering, amplification, and digitization. The buffer transistor needed to transform the impedance both increases the noise and footprint of each pixel. Further, this noise and any other high-frequency noise (up to ~1 MHz) from the electrode, pixel circuitry, and environment is aliased into the neural frequency range (1 Hz–10 kHz). A filter and amplifier at each pixel could remedy this aliased noise but would prevent high density recording due to their size. In this work, we reduced the impact of aliased noise by developing a multiplexed electrode design that utilizes an integrating amplifier needed for current sensing rather than voltage sensing (Supplementary Fig. 3b). Since current noise from the brain has a 0 A average, the integrating amplifier decreases high-frequency noise that would be later aliased into frequencies of interest lessening the need for a low pass filter at each pixel. Further, this circuit design does not need a high input impedance, so the noisy buffer transistor (T1) was removed, leaving only the multiplexing transistor (T2). Indeed, our previous work has shown that passive arrays without multiplexing can capture comparable neural signals utilizing ultra-sensitive, current-sensing circuit designs. Neural decoding accuracy and error were indistinguishable between current and voltage recordings. There are also commercially available integrated circuits that provide ultra-low noise measurements of multiplexed currents from up to 256 channels simultaneously. Combining electrodes that incorporate 32:1 multiplexing will eventually yield electrode arrays with up to 8192 recording channels with less than 300 wires, suggesting that the current-sensing design is highly scalable. Finally, our current-sensing, multiplexed design can enable increased device lifetime with simple polymeric encapsulation by eliminating any DC bias from the active circuitry on the array.

Our soft and ultrathin neuroelectronic array can be precisely delivered into the brain through syringe injection, which is suitable for a minimally invasive implant^34^. For injection, we used a transparent capillary tube with an inner diameter of 400 μm and an outer diameter of 600 μm (Drummond Scientific, capillary micropipettes) to load the device, which is 300 μm wide. The transparency of the tube allowed us to see how the device moved inside it. We loaded the device, electrode side first, while the device was floating inside the PBS solution. We then flipped the capillary tube for the injection. Loading the pad side first was found to be more difficult since the natural width of the pads was larger than the inner diameter of the tube. Upon loading, the meshed I/O pads spontaneously rolled in the transverse direction (normal to the tube) to fit inside the tube (Supplementary Fig. 4), which would unroll to their full size once ejected out. We injected the soft neuroelectronic array into 0.6% brain phantom (agarose gel) to explore the precision of insertion. We first moved the electrode part close to the end of the syringe tube and then inserted the tube to the desired depth in the phantom. To properly control the device injection into the target region, we leveraged a syringe pump and a motorized stand (Supplementary Fig. 5). The syringe pump injected water, which pushed the device downward with respect to the syringe tube, while the motorized stand held the syringe and simultaneously moved it upward with the same velocity as the water ejection from the tube, making the device remain at the same position relative to the phantom. In our setting, we used a flow speed of 3 mL/min for the syringe pump and an upward movement of 20 mm/min for the motorized stand. The injection process is demonstrated in a high-definition video recording (Supplementary Video 1). This level of control can achieve the desired delivery with less than 100 μm inaccuracy, allowing for implantation with high precision. Utilizing intraoperative imaging techniques and microelectrode recording (MER) can significantly improve probe placement precision by offering real-time guidance and accurate targeting during the implantation process, approaches that are frequently used in Stimwave and deep brain stimulation (DBS) electrodes^35–37^.

### Fabrication and device bench testing

**Fig. 2:**
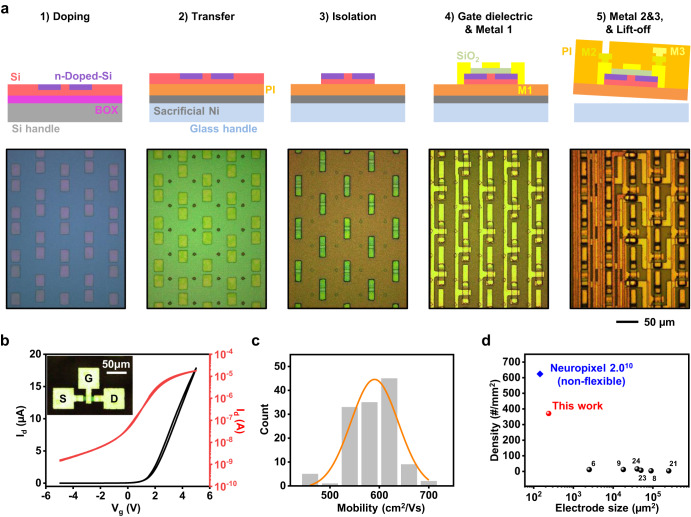
Fabrication and electrical performance of soft, high-density neuroelectronic arrays. **a** Cross-section schematics (top) and optical images (bottom) of key fabrication steps, including doping, transfer printing, isolation of n-doped Si NMs, gate dielectric deposition and metal 1 patterning for input selects, metal 2 & 3 patterning for output signals, and lift-off as the final step. **b** Representative I-V characteristics of a Si NM transistor used in the device. Inset: Optical image of a representative test transistor. **c** Mobility histogram of 256 transistors with an average value of 583.7 ± 48.3 cm^2^/Vs. **d** Comparison of this work, other flexible bioelectronics, and Neuropixels in electrode size and density.

The schematics and optical images (Fig. 2a) show five critical steps during the fabrication process. Briefly, the fabrication began with n-doping of Si (thickness ~140 nm) on a silicon-on-insulator (SOI) wafer (Supplementary Fig. 6). Then, we undercut the buried oxide (BOX) layer in the SOI and transfer-printed n-doped Si nanomembranes onto a flexible PI substrate, which was pre-spin-coated on Ni surface with a glass handle, with the Ni film serving as a sacrificial layer for the eventual device release at the end of the process. After isolating the Si nanomembranes using conventional photolithography and dry etching, plasma-enhanced chemical vapor deposition (PECVD) of silicon dioxide (SiO_2_) created the gate dielectric. A series of sputter deposition and conventional photolithography steps completed the metal definition for the transistor channels, row selects, and column outputs. Spin coating deposited thin PI layers for interlayer dielectrics. After the last PI coating encapsulated the device, reactive ion etching (RIE) removed unnecessary parts and achieved the desired device profile, including micro-meshed I/O pads (Supplementary Fig. 7). Finally, a Ni etchant removed the Ni sacrificial layer at the bottom to release the device from the glass handle. To completely remove residues of the Ni etchant from the array, the devices went in and out of clean deionized (DI) water five times with a pipette. A more detailed procedure can be found in the “Methods” section.

We then performed bench testing of the soft neuroelectronic array. The I-V characteristics of these Si transistors typically exhibit threshold voltages of ~1.5 V and an ON/OFF ratio of ~10^5^ with an inset showing a test transistor (Fig. 2b). A histogram of 256 transistors shows high mobility of 583.7 ± 48.3 cm^2^/Vs (Fig. 2c), demonstrating their electrical performance and high uniformity. Since this transistor acts as a switch, any variation in the process does not affect the accuracy of the electrical recording. We also investigated the effect of different etch-hole pitches on the performance of the Si device layer. These 3-μm-diameter-etch-holes provided a pathway for hydrofluoric acid (HF) to undercut the buried oxide layer, allowing the transfer of the Si device layer to the flexible substrate. When these holes overlapped with the doped-Si areas, the electrical performance and yield of the transistors decreased due to the partial loss of Si, presumably due to the small footprint of the transistors. By matching the etch-hole pitch with the Si transistor pitch (40 μm) and avoiding overlap, we could achieve transferred transistors with both high performance and yield (Supplementary Fig. 8). We also compared the device performance before and after the release process and observed no significant difference (Supplementary Fig. 9). The mechanical robustness of the flexible arrays is also crucial for both the device utilization and in vivo applications. After 10,000 bending cycles to a radius of 4 mm, we observed no significant performance degradation in its electrical performance, nor any visual damage from the device, demonstrating its flexibility and reliability (Supplementary Figs. 10 and 11). The slight shift of threshold voltage comes from sample-to-sample variation in different batches of transistor fabrications. We compared our work to existing soft CMOS-based bioelectronics, showing that our device demonstrated 2–3 orders of magnitude improvement in electrode density and size (Fig. 2d) with a similar level of SNR (Supplementary Table 1). These metrics are also close to those of Neuropixels, which is a non-flexible, Si-based device.

### Noise study and data acquisition system

**Fig. 3:**
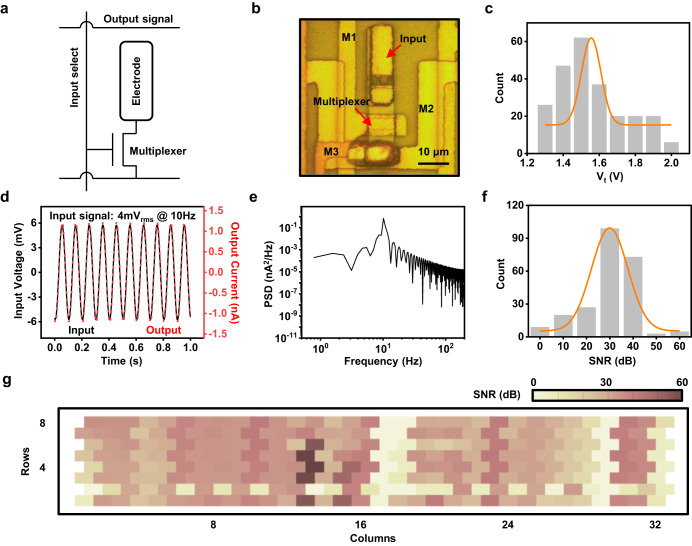
Bench testing of soft, high-density neuroelectronic arrays. **a** A circuit diagram for a pixel in the active array, having a Si transistor as a multiplexer. **b** Corresponding optical image of the circuit diagram in A from a microscope. **c** V_t_ histogram from 256 transistors with an average value of 1.56 ± 0.19 V. **d** Bench recording of a 10 Hz, 4 mV_rms_ sine-wave input voltage (black) and corresponding output current (red). **e** Power spectra density (PSD) of the recorded sine-wave output in (**d**). **f** SNR histogram of a 256-ch array with an average value of 30.1 ± 10.4 dB. **g** SNR heatmap of a 256-ch array.

We measured extracellular neural currents (rather than voltage) with our soft neuroelectronic array by integrating charge over the sampling period. A circuit diagram (Fig. 3a) and optical image (Fig. 3b) show that each electrode was coupled with a multiplexer. This multiplexer consists of one Si transistor of a small Si footprint, with 35 μm channel width and 5 μm channel length. We tested two more channel lengths, 7 μm and 10 μm, but 5 μm showed the best results in terms of mobility, yield, and ON/OFF ratio (Supplementary Fig. 12). The array showed an average threshold voltage of 1.56 ± 0.19 V (Fig. 3c), showing good uniformity. In vitro testing demonstrated the device’s ability to record local field potentials frequency ranges with sufficient SNR to decode neural signals. Our experiments suggested that current-sensing multiplexed acquisition circuits may have lower noise than voltage-sensing ones while providing similar levels of information from the brain. In vitro tests of passive electrodes connected to the current-sensing system recorded noise of 16.4 ± 10.0 pA_rms_ (noise bandwidth 1–200 Hz) and high SNR of 70 ± 9.8 dB using a 250 μV_rms_, 10 Hz sine wave (Supplementary Fig. 13). This noise value is near the noise floor of the AFE0064 current recording device, indicating that the subsequent data acquisition (DAQ) circuitry introduced very little noise. We used SNR to compare performance since noise or gain measurements were not comparable across sensing modalities (i.e., current vs. voltage). Next, we integrated the data acquisition system with 1-transistor active electrodes in the soft neuroelectronic array. In vitro testing, where the entire device was submerged in PBS solution, and a sine wave (4 mV_rms_, 10 Hz) was applied, demonstrated little to no distortion by the electrodes (Fig. 3d). The power spectral density of the recorded signal showed a peak at 10 Hz with a 1/f decay as expected (Fig. 3e). We also demonstrated the capability of recording a 1 kHz sine wave (Supplementary Fig. 14) for higher-frequency recordings. The distortion and power spectral density were similar to that at 10 Hz. The histogram of SNR from all 256 microelectrodes shows electrical performance, with an average value of 30.1 ± 10.4 dB (over 1–200 Hz, with input = 4 mV_rms_, 10 Hz sine) with a 92% yield (Fig. 3f), much higher than our previous active arrays with 2-transistor voltage-sensing design (22 dB; 2–100 Hz). The SNR spatial distribution of all electrodes with respect to their actual position also demonstrated good uniformity (Fig. 3g and Supplementary Fig. 15). The average noise of the combined current-sensing system and 1-transistor active electrodes was 2.7 ± 3.4 pA_rms_ (1–200 Hz). We developed a second current-sensing circuit with the ability to record at higher sampling rates with similar noise and SNR performance. In vitro tests with 1-transistor active electrodes in the soft neuroelectronic array show SNR of up to 60 dB (over 2–4500 Hz, with input = 5 mV_rms_, 1 kHz sine) and noise as low as 32.3 pA_rms_ (over 2–4500 Hz; Supplementary Table 2) which relates to ~6 μV_rms_ noise. The noise and SNR values for both circuits are sufficient to acquire local field potentials and possibly higher-frequency range signals, and are dramatically better at high frequencies than previous 2-transistor voltage-sensing designs.

### In vivo validation in rats

**Fig. 4:**
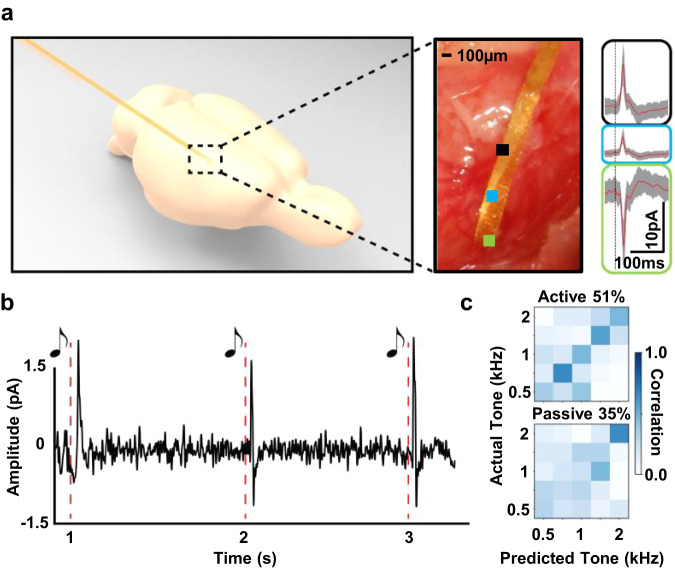
In vivo results of a soft, high-density neuroelectronic array. **a** Schematic of the device implanted on the surface of rodent primary auditory cortex (left), averaged responses from three independent channels, and annotations of the electrode positions in the array (right). **b** Single-trial neural responses to three successive tone stimuli from the tone task. Data were filtered from 1–100 Hz with a 6th order Butterworth filter. **c** The soft neuroelectronic array achieved a decoding accuracy of 51% compared to 35% accuracy from the passive array in a tone discrimination task.

In vivo testing of soft neuroelectronic arrays from multiplexed, current-sensing electrodes (Fig. 4a) in rats yielded readily visible, single-trial auditory evoked responses (Fig. 4b) and decodable neural signals (Fig. 4c). We completed an acute epidural recording over the A1 auditory cortex in an anesthetized rat following established procedures in our lab^27^. To better compare our 1-transistor active devices to both traditional passive voltage-sensing electrodes and existing knowledge of the auditory cortical surface, for the initial in vivo validation, we laid the device on the cortical surface, not injecting the device into the cortical layer. We connected the device to the DAQ system with an anisotropic conductive film (ACF) cable simplifying the connection process for post electrode implantation.

Thirteen different tones (0.5–32 kHz, 0.5 octave spacing, 50 ms in duration, 2 ms cosine-squared) at 70 dB were played over a speaker inside a sound-attenuated chamber while recording from the cortex. This experimental design allowed for single-trial tone decoding of tone responses. Tone decoding accuracy was comparable to passive electrode recordings from the same animal, but the active array had improved tone decoding performance for a subset of tones (0.5–2 kHz). We decoded this subset of tones with higher accuracy (51%) using the 1-transistor array compared to the passive array (35%). This increase in performance was expected because the particular design we tested had higher electrode density over a small area, which is 2300 × 300 μm^2^ of cortex compared to that of the passive arrays.

Due to limitations in the DAQ design, we were not able to record single-unit activity from our array, but electrode surface area and noise indicate that this is achievable. Overall, these arrays demonstrated the ability to record in vivo neural signals with similar or increased performance compared to standard passive electrode arrays.

## Discussion

In summary, we developed a soft, high-density neuroelectronic array with the ability to record decodable neural signals and a design that supports further upscaling. On-site multiplexing using Si nanomembrane transistors greatly reduced the number of external wire connections to record from all 256 electrodes to only 40. The narrow array width combined with the rollable contact design demonstrated the potential for the device to be deliverable through syringe injection, yielding a minimally invasive tool for chronic neural recording in deeper regions of the brain. We utilized a current-sensing, 1-transistor architecture which reduced the footprint and improved the noise performance of the active electrode compared to previous 2-transistor devices by mitigating aliased noise introduced by multiplexing. The device also pairs well with commercially available integrated circuits that provide ultra-low noise measurements of multiplexed currents allowing significant scalability. This design could also increase the device lifespan and allow for simple polymeric encapsulation strategies due to the 0 V DC bias. Previous work demonstrated that device lifespan dramatically decreases when held at a DC bias needed for a 2-transistor electrode design. While this issue can be solved through alternative encapsulation strategies, such as using thermal silicon dioxide, they leave the device less flexible. Using this electrode design, we recorded single-trial-decodable neural signals with comparable or improved information content than standard passive micro-electrocorticographic arrays.

Future work will also be devoted to achieving chronic in vivo recording capabilities for at least several months, if not years, to fully enable other applications. The great flexibility of the array will be able to lessen the damage to the surrounding tissue after implantation and decrease the subsequent immune response, increasing the longevity of neural recordings. While the soft neuroelectronic arrays here are fabricated from conventional photolithography in University cleanrooms, we envision leveraging commercial foundry and advanced post-processing for future generations. Future efforts can leverage massive multiplexing to allow for larger-scale throughputs with thousands of channels covering a large area with a high density of electrodes and few interfacing wires. With the improvements in the sampling rate of the data acquisition system, future devices will also be able to record single-unit responses with multiplexing and spatiotemporal analysis. Developing approaches to inject fully-connected devices will also be highly impactful future work. Overall, the soft neuroelectronic array demonstrated here can enable simultaneous recording from hundreds of small, soft electrodes while utilizing a limited number of interfacing wires, allowing scientists to investigate distributed brain functions that arise from the coordinated activation of neuronal assemblies in the brain.

## Methods

### Fabrication of the soft, high-density neuroelectronic array

The fabrication began with PECVD deposition of 300-nm-thick SiO_2_ with 75 nm/min on SOI wafer with 175-nm-thick Si and 1-µm BOX (SOITEC). Conventional photolithography and RIE patterned doping profile. The RIE condition was 200 W for radio frequency (RF) _1_, 150 W for RF_2_, 10 mT, 40 sccm CHF_3_ for 2 min. 10:1 buffered oxide etchant (BOE) removed oxide residues to complete doping profile etching. After removing the photoresist (PR) by sonication in acetone, an RCA process cleaned the samples before doping. Then, the samples were spin-coated with spin-on dopant (P451; Filmtronics) at 1500 rpm for 20 s and soft baked for 5 min at 180 °C. A doping furnace doped the samples for 5 min at 900 °C. After doping, 49% HF acid removed the oxide mask completely. For transfer printing^38^, photolithography patterned periodic 3-µm-diameter dots, then RIE etched top Si layer. Soaking in 49% HF for ~20 min lifted the top Si layer, and PDMS stamp transferred the layer onto PI layer, which was pre-spin-coated on 100 nm-thick-Ni on a glass substrate. Then, the transferred samples were baked for 70 min at 250 °C in N_2_ ambient atmosphere. Conventional photolithography and RIE steps isolated doping patterns to create individual NMOS nanomembranes. The RIE condition was 200 W for RF_1_, 400 W for RF_2_, 20 mT, 40 sccm SF_6_ for 90 s. After removing PR in hot acetone for 5 min for a brief cleaning (the RCA process cannot be used because of the PI layer underneath), PECVD deposited 100-nm-thick SiO_2_ at 200 °C as the gate dielectric. A photolithography step and an etching using 10:1 BOE created vias, sputter-deposited 5 nm of Cr and 100 nm of Au for metal 1, which is channel creation and column select. By stacking different metal layers with sputtering and patterning interconnects with conventional photolithography, we achieved three metal layers with 1-µm-thick PI as interlayers to separate each 100-nm-thick metal layer. Then, the last 1-µm-thick PI encapsulated the whole device and defined the electrodes. The RIE condition for etching PI was 200 W for RF_1_, 150 W for RF_2_, 20 mT, 20 sccm O_2_, 2 sccm CHF_3_ for 3 min. Then, photolithography and RIE patterned profile of the whole device. The RIE condition for profile etching was 400 W for RF_1_, 200 W for RF_2_, 20 mT, 20 sccm O_2_, 2 sccm CHF_3_ for 15 min. Finally, the device was put inside Ni etchant for release. To wash away all Ni etchant, the devices went in and out of clean DI water five times with a pipette. All materials used for the device are considered biocompatible for implanted biomedical devices^39,40^.

### Bench testing

We performed bench testing measuring noise and SNR to evaluate the performance of the soft, high-density neuroelectronic array and DAQ system both separately and combined. For the DAQ noise recording, we connected standard gold passive electrodes (Dyconex) to the DAQ and placed the electrode contacts in saline along with a grounded Pt wire. Next, we applied a sine wave with properties similar to that of a neural signal (input = 720 μV_rms_, 10 Hz sine) to the Pt reference wire to determine SNR. SNR was calculated as the output of the system with this neural-like signal divided by the output with a grounded input (noise). For the combined system noise and SNR, we replaced the passive electrodes with soft, high-density neuroelectronic array and repeated the tests using larger sine waves with different frequencies (input = 4 mV_rms_, 10 Hz sine; 5 mV_rms_, 1 kHz sine). We used a microscope to manually align pads of ACF to the ones of devices.

### Recording system

We acquired neural currents using a custom multiplexed recording system based on an ultra-low noise current-sensing amplifier (AFE0064, Texas Instruments). The neural signal was measured as current or charge over time. The 256-ch electrode array connected the 32 multiplexed column outputs and 8 row-select inputs through a 45-pin ZIF connector (Hirose Electric Co., LTD.) to an interface board. A current-sensing amplifier measured electrode current rather than voltage, which we have shown to have comparable or improved SNR in passive electrode recordings. The AFE0064-integrated circuit integrated the current, converting the readout to voltage from 64 channels simultaneously using 64 current integrator circuits with correlated double-sampling. We constructed a separate current-sensing circuit to increase the sampling rate and further reduce noise for optimal neuronal recording. This circuit contains a trans-impedance amplifier that converts current to voltage rather than the integrator used by the AFE0064. The circuit can support a 256-ch electrode array connected to 16 multiplexed column outputs and 16 row-select inputs through a 45-pin ZIF connector (Hirose Electric Co., LTD.) to an interface board. Both circuits lead into the remote data acquisition system (Supplementary Fig. 16), where the differential analog outputs of the amplifier were buffered by four dual op-amps in a unity gain configuration (OPA2376, Texas Instruments). The differential analog outputs of the amplifier were converted to digital using two 18-bit analog-to-digital (A2D) converters (PXI-6289, National Instruments), sampled at 625k samples per second (sps). The final sampling rate was 390 sps per electrode channel; however, this can be further optimized with future software revisions.

### Surgical procedure for anesthetized recordings

The Duke University Institutional Animal Care and Use Committee approved all animal procedures. Experiments were carried out in a sound-attenuation chamber. We anesthetized female Sprague Dawley rats aged 4–6 months using Isoflurane (Patterson Veterinary), administered through a vaporizer (Absolute Anesthesia) throughout the duration of the recording. We mounted the rodent’s head in a custom head-holder orbital clamp to leave the ears unobstructed. Next, we cut longitudinally along the midline to expose the skull and retracted the left temporalis muscle. Using a rotary tool, we created a craniotomy on the left temporal skull (6.5 × 6.5 mm). Finally, we placed a sterilized electrode array over the core auditory cortex using vascular landmarks epidurally. After implanting the device, we connected our device to the DAQ system using an ACF cable, clamp and PDMS to apply even pressure across the I/O pads (Supplementary Figs. 17 and 18)^41^. We adjusted the placement of the electrodes by identifying the primary auditory cortex using recorded evoked responses to stimulus clicks and tones. We drilled one screw hole and placed a bone screw in the skull over the opposite hemisphere from the recording site as a reference for the electrode. All recordings were completed in a sound-attenuated chamber. Custom MATLAB code generated acoustic stimuli through an NI PXI-6289 DAC card. A free-field speaker (CR3, Mackie) delivered the stimuli and was calibrated to have a flat output over the frequency range used. Neural responses to tone pips of 13 frequencies (0.5–32 kHz, 0.5 octave spacing, 50 ms in duration, 2 ms cosine-squared) at 70 dB sound pressure level (SPL) were collected for tonotopic mapping. We presented tones in a pseudorandom sequence once per second; each tone was repeated for 30 trials. Responses to click stimuli (0.2 ms in duration, 70 dB SPL, 1.25 Hz, 120 repetitions) were also recorded for each electrode. Clicks contain a broad spectrum of tones, thus exciting a large portion of the primary auditory cortex. We euthanized the animal after the procedure through injection of Euthasol (intraperitoneal, 250 mg/kg).

### In vivo tone decoding

We used the linear classifier to predict tones given array responses^42^. We separated responses from the field potential at each channel, then bandpass filtered (2–100 Hz) and windowed following each tone response (5–80 ms). A channel was removed from the classifier if its responses contained more than 5% outliers. We determined tone decoding accuracy (the proportion of successfully classified trials) and the average error of classification, the octave difference between the predicted and true tone.

### Supplementary information

Supplementary Information

Supplementary Video 1

## Data Availability

All data generated or analyzed during this study are included in this published article and supplementary information files.

## Code availability

The underlying code for this study is not publicly available but may be made available to qualified researchers on reasonable request from the corresponding author.
