# Supplementary material for: A soft, high-density neuroelectronic array
Source: Npj Flex Electron. 2023 Aug 22;7(1):40. doi: 10.1038/s41528-023-00271-2 (PMC10487278; doi:10.1038/s41528-023-00271-2)
Supplement: Supplementary file 1 — Supplementary Information [file 41528_2023_271_MOESM1_ESM.pdf]

Supplementary Information

A Soft, High-Density Neuroelectronic Array

Kyung Jin Seo[1], Mackenna Hill[2], Jaehyeon Ryu[1,3], Chia-Han Chiang[2], Iakov Rachinskiy[2], Yi Qiang[1], Dongyeol Jang[1], Michael Trumpis[2], Charles Wang[2], Jonathan Viventi[2,*], and Hui Fang[1,3,*]

[1]Thayer School of Engineering, Dartmouth College, Hanover, NH 03755

[2]Department of Biomedical Engineering, Duke University, Durham, NC 27708

[3]Department of Electrical and Computer Engineering, Northeastern University, Boston, MA 02115

These authors contributed equally to this work: Kyung Jin Seo, Mackenna Hill

*Email: hui.fang@dartmouth.edu (Hui Fang), j.viventi@duke.edu (Jonathan Viventi)

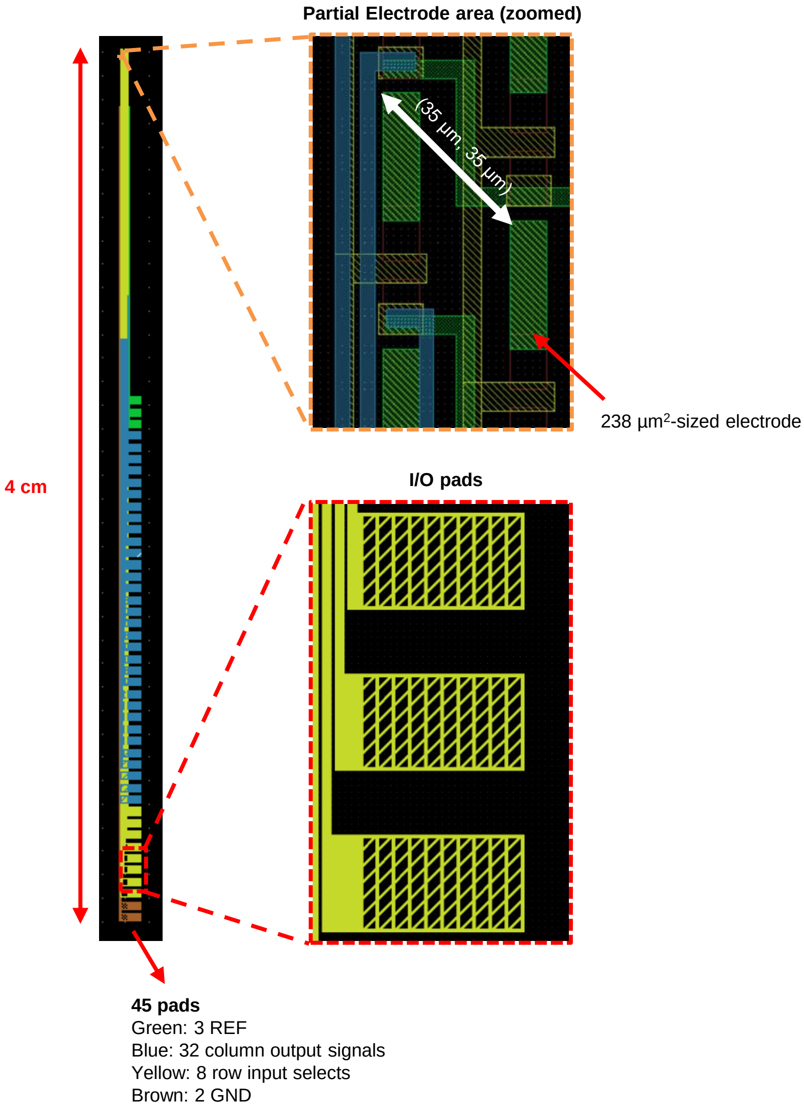

Supplementary Figure 1. Design of the soft, high-density neuroelectronic array. The electrode area is 300 x 2,300 µm^2. Individual meshed I/O pads are 300 x 500 µm^2 with pitch of 500 µm.

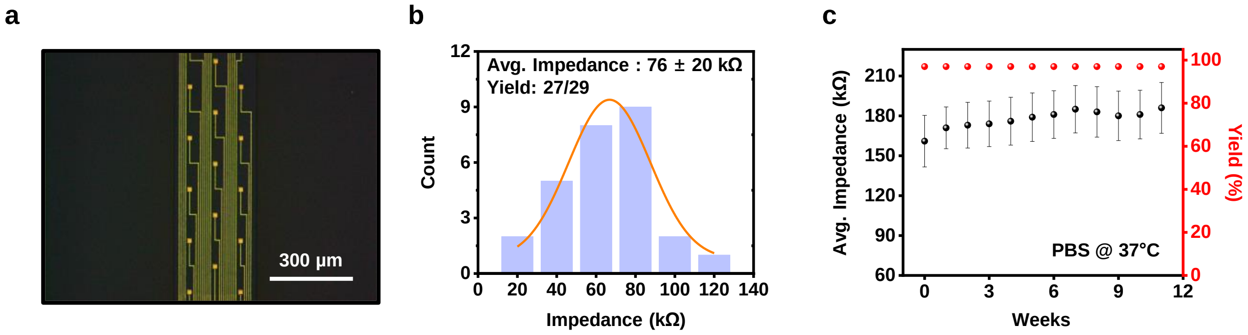

Supplementary Figure 2. Performance of passive arrays. a An optical image of a 29-ch passive array similar in size to the soft, high-density neuroelectronic array. **b** electrical impedance histogram measured at 1kHz. **c** soaking test for three months in PBS.

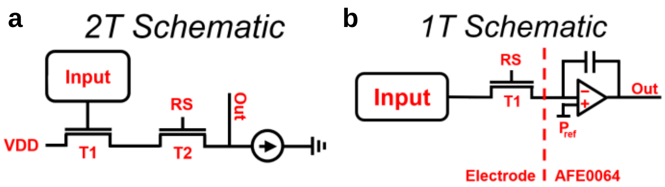

Supplementary Figure 3. Multiplexing Supporting Architecture. a Our standard 2T design uses a buffer transistor (T1) and a multiplexing transistor (T2) to record multiplexed neural signals (Chia-Han Chang, 2020). **b** 1T multiplexed, current-sensing design with increased SNR. Neural currents from the electrode (Input) are integrated on a small capacitor within the ultra-low noise current-sensing amplifier (AFE0064). A single transistor (T1) at each electrode is periodically switched on to record from the brain using an external amplifier.

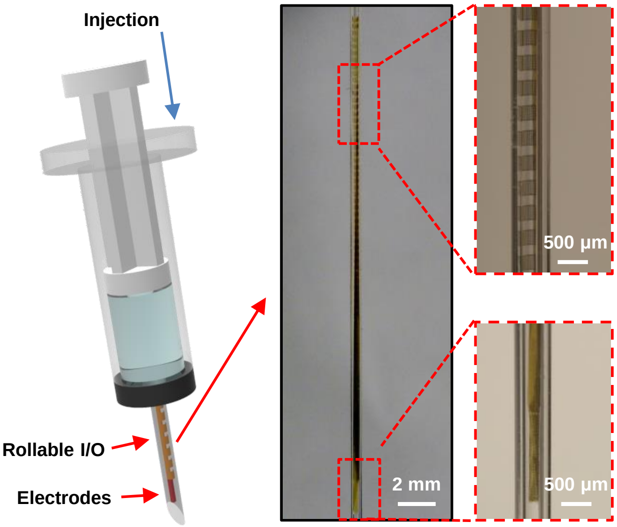

Supplementary Figure 4. Schematic and optical images of an array loaded inside a capillary tube (I.D.: 400 µm, O.D.: 600 µm).

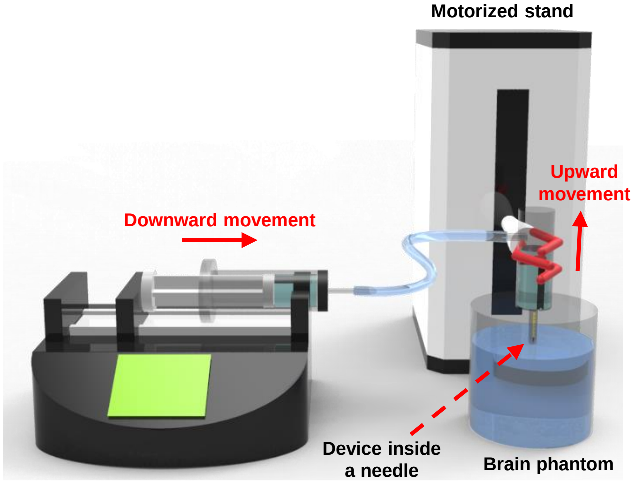

Supplementary Figure 5. Injection setup.

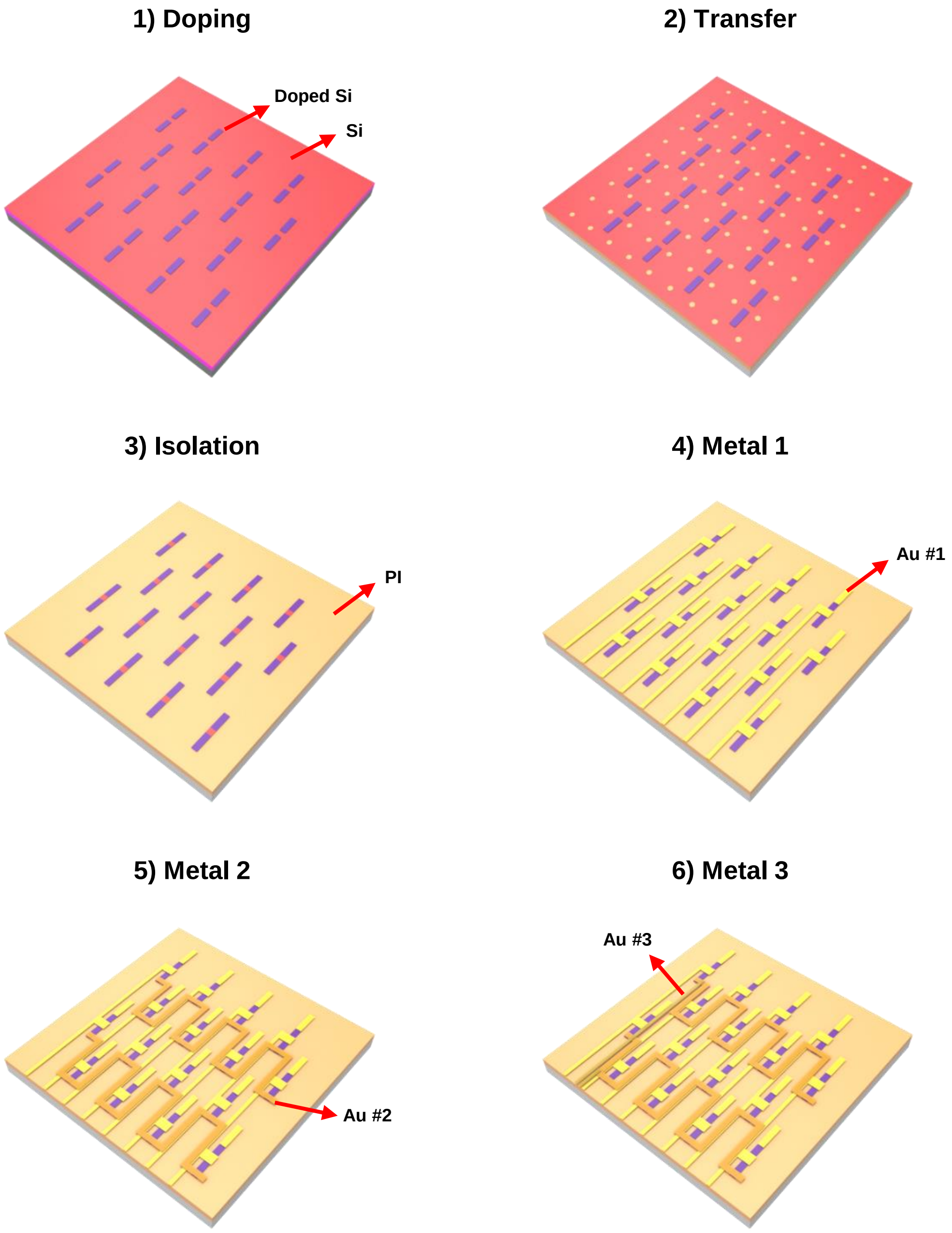

Supplementary Figure 6. Key fabrication steps of soft, high-density neuroelectronic array.

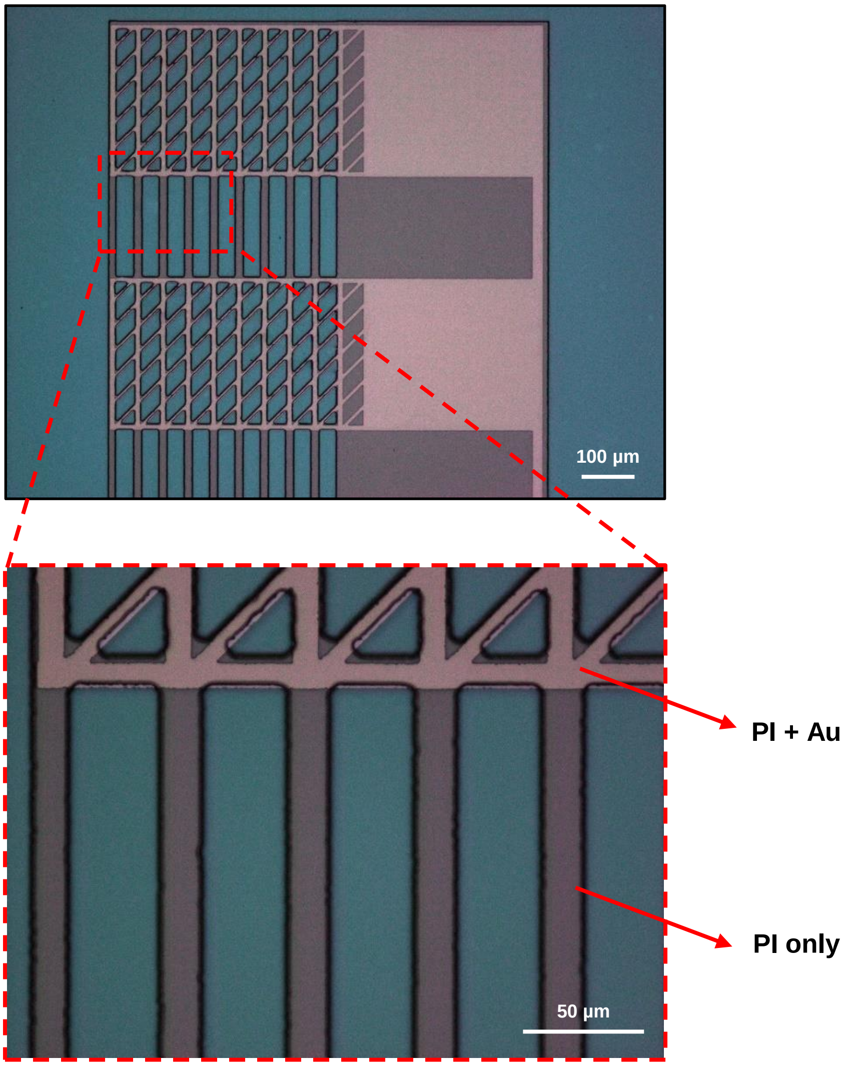

Supplementary Figure 7. Optical images of micro-meshed, rolling I/O pads.

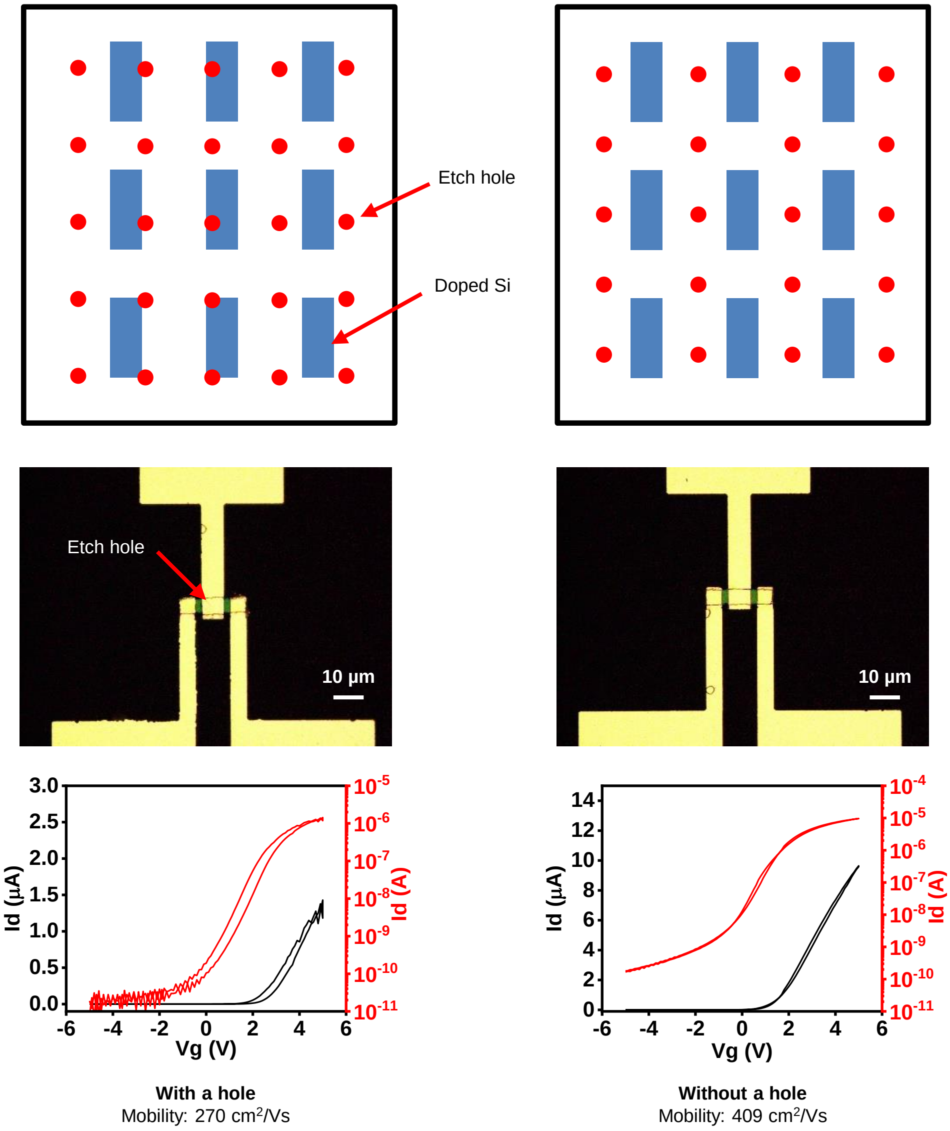

Supplementary Figure 8. I-V characteristics of transistors with different etch hole pitch.

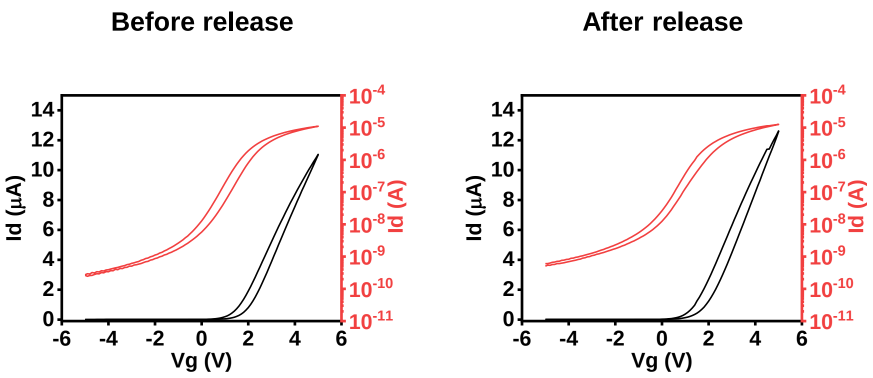

Supplementary Figure 9. Typical I-V characteristics of transistors before and after the release process.

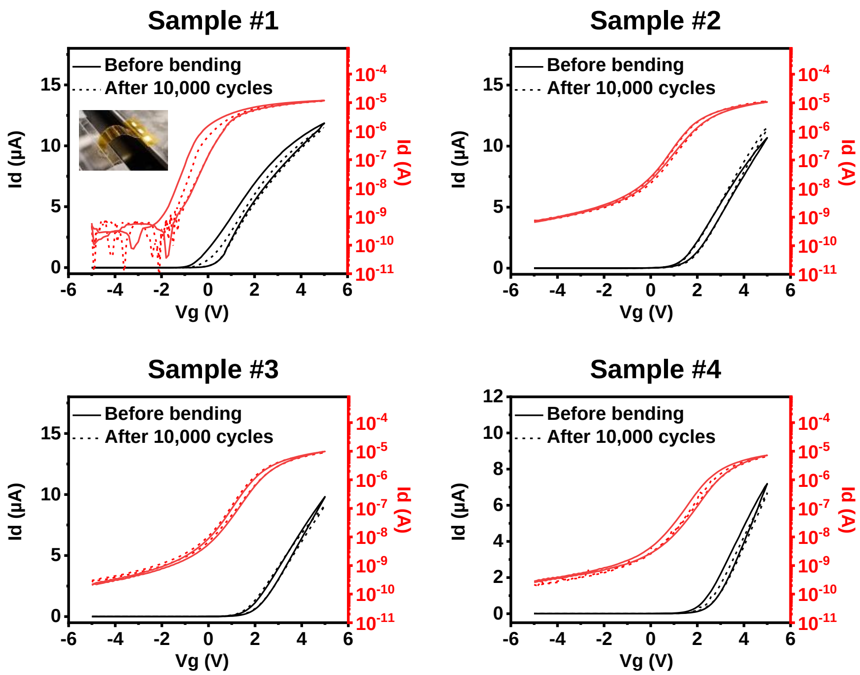

Supplementary Figure 10. Mechanical bending tests of a single transistor with 2cm-long, 5μm-wide interconnects.

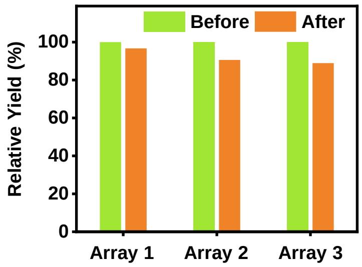

Supplementary Figure 11. Mechanical bending tests of 256-ch soft, high-density neuroelectronic array with 10,000 bending cycles using 4mm-bending radii. SNR before and after bending with an input of 65mV$_{rms}$, 1kHz sine wave recorded at 10kHz. Changes in SNR greater than 20dB indicated failed electrodes.

5 µm channel length   7 µm channel length   10 µm channel length

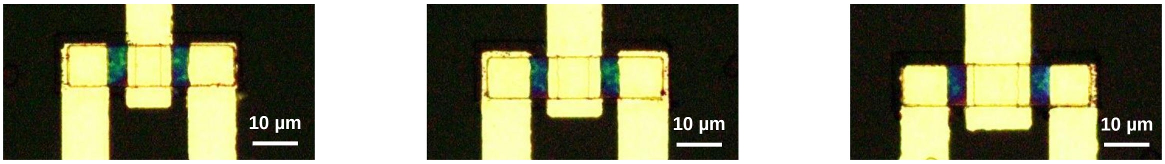

Channel Length (µm)	Avg. Mobility (cm^2/Vs)	On/off ratio
5	646	10^5
7	634	10^5
10	536	$10^{4.5}$

Supplementary Figure 12. I-V characteristics of transistors with different channel lengths.

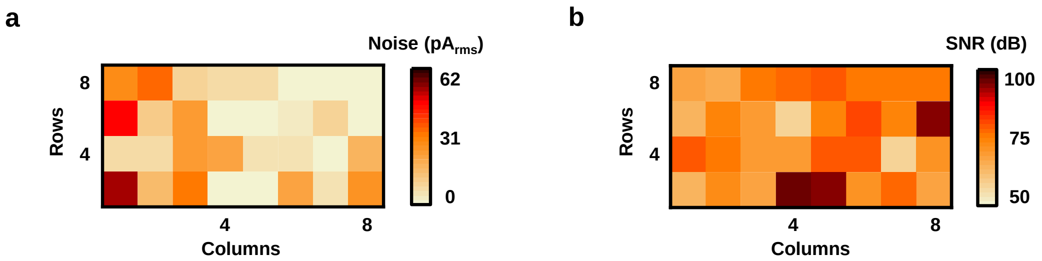

Supplementary Figure 13. Performance of a 32-ch passive electrode connected to the current-sensing system. a Noise heatmap, showing noise of 16.4 ± 10.0 pA_{rms} (bandwidth 1 – 200Hz). **b** SNR heatmap, showing SNR of 70 ± 9.8 dB using 500 μV_{rms}, 10 Hz sine wave.

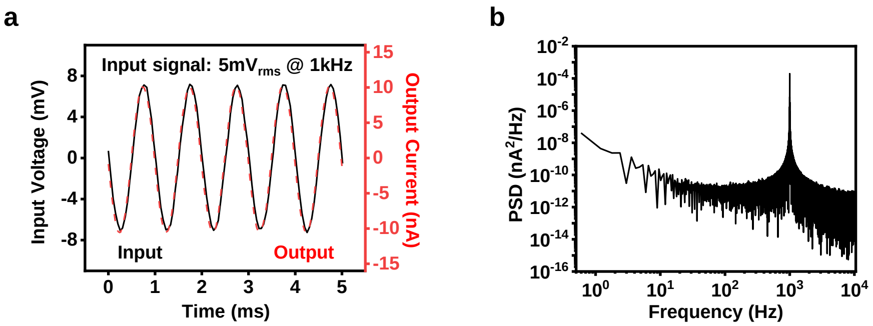

Supplementary Figure 14. Recording and noise analysis. a Bench recording of a 1 kHz, 5 mV_{rms} sine wave as normalized input voltage (black) and corresponding output current (red). **b** Power spectra density (PSD) of the recorded sine-wave output in **a**.

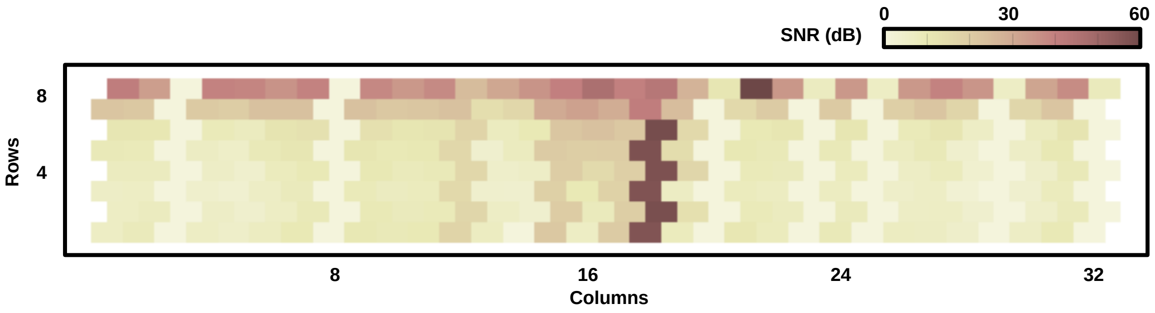

Array #1; SNR: 15.8 ± 13.0 dB, Yield: 80%

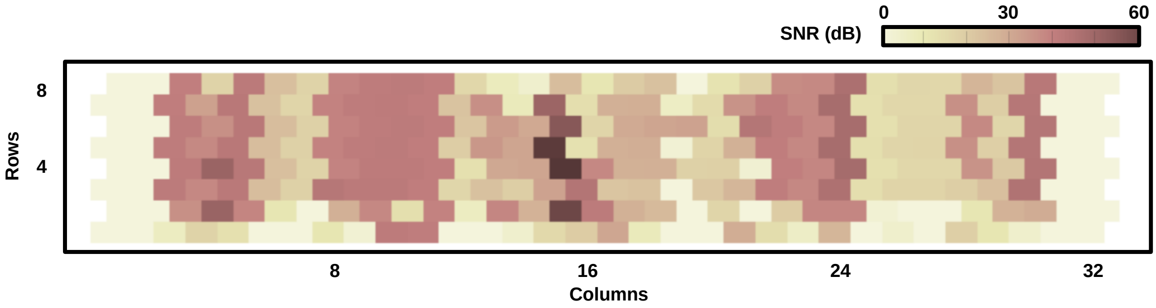

Array #2; SNR: 28.0 ± 13.8 dB, Yield: 79%

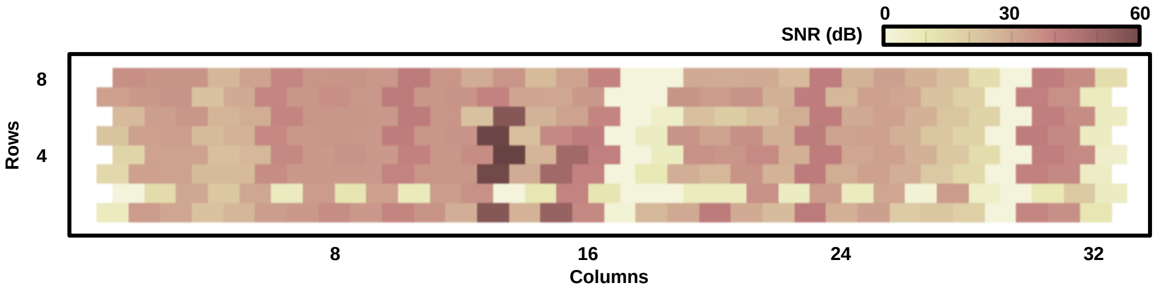

Array #3; SNR: 30.1 ± 10.4 dB, Yield: 92%

Supplementary Figure 15. SNR recordings from different neuroelectronic arrays. Non-working electrodes were not included in mean SNR calculations. Non-working electrodes were determined as electrodes with less than 10 dB SNR and the number of non-working electrodes is reflected in the yield.

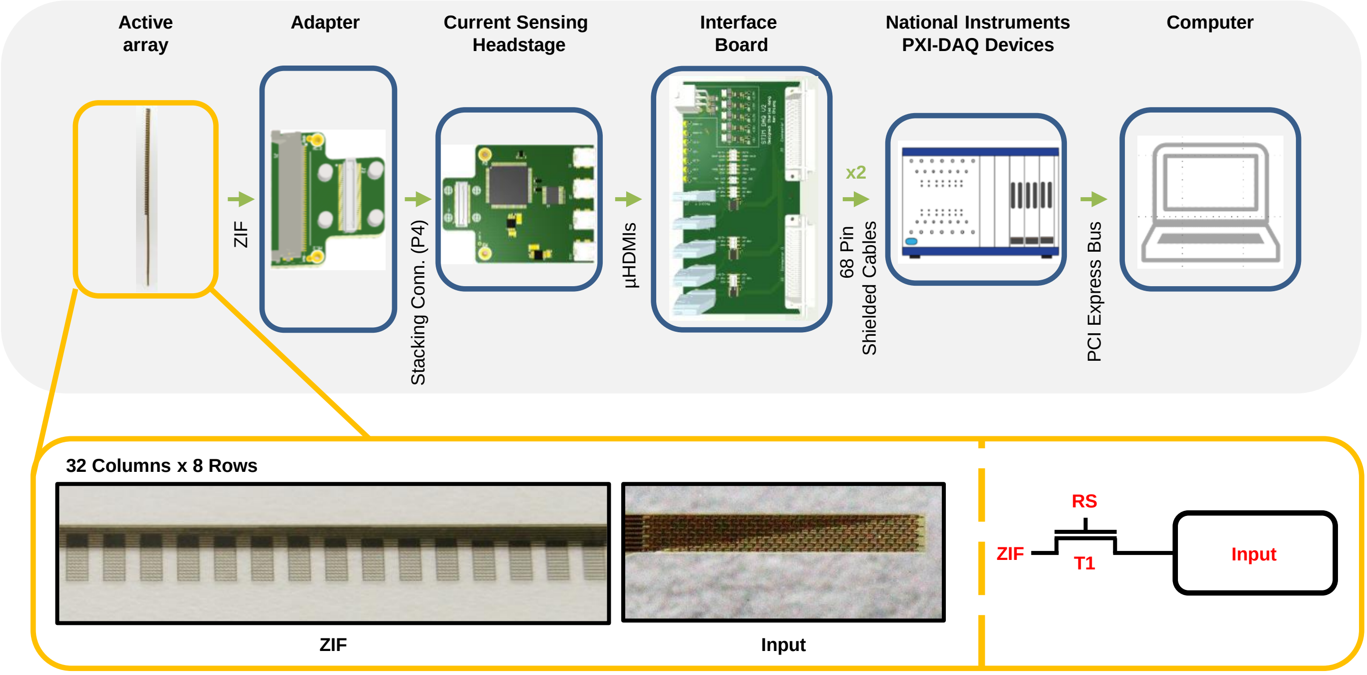

Supplementary Figure 16. Illustration of the information flow from the brain to the computer indicating each stage and connecting cables. The breakout shows the neuroelectronic array contacts, ZIF connector, and schematic.

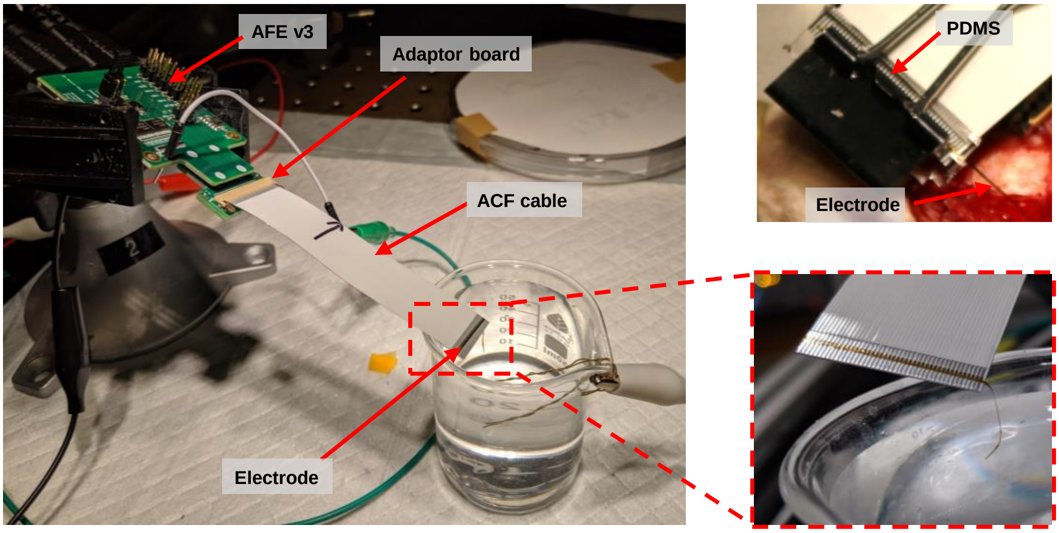

Supplementary Figure 17. Recording setup. (Left) Recording setup from electrode, broke out in the bottom right, to the ACF cable, adaptor board, and finally AFE current recording headstage. (Top-right) Clamp used to keep electrode pads aligned and connected to ACF cable.

1. Device Implant

2. ACF Cable Connection

3. PDMS

4. Securing Contact

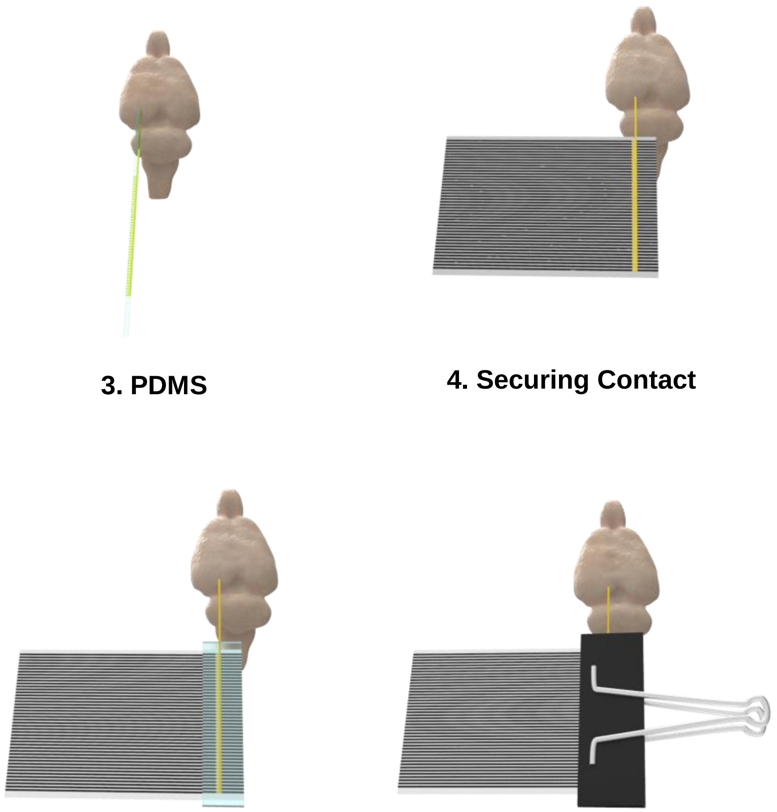

Supplementary Figure 18. Insertion steps. Implant the device with syringe into the brain. Next, remove syringe and carefully place the device on to ACF cable, and place PDMS slap on top of device. Finally, secure both PDMS and ACF cable for better contact.

Number of electrodes	Electrode size (μm×μm)	Coverage (mm×mm)	Density (# of electrode/mm^2)	Multiplexing Ratio	SNR (dB)	Ref.
1152	50×50	14×7	11.75	1	-	Kaiju[6]
12	200×200	10.8×7	0.16	1	28.8	Zhang[14]
128	20×20	2×0.33	192	1	<20	Fu[11]
5120	12×12	0.82×10	624	1	54.9	Steinmetz[10]
64	250×200	3×3	7.11	4	-	Yu[23]
196	200×200	3.5×3.5	16	6.75	-	Escabi[24]
256	300×300	-	-	16	64	Huang[13]
1008	100×180	9×9.24	12.12	16	36.8	Chiang[9]
360	300×300	10×9	4	18	42.5	Viventi[8]
396	500×500	9.5×11.5	3.62	22	42	Fang[21]
1024	50×50	-	-	32	41.9	Fernandez[25]
256	34×7	2.3×0.3	371	8	30.1	This work

Supplementary Table 1. Comparison of performance between other devices and this work. Multiplexing ratio refers to the number of individual electrodes measured on the same output wire and demonstrates scalability. Traditional passive electrodes one output wire for each electrode and thus a multiplexing ratio of 1.

**Noise (2 – 4,500 Hz)**	**32.3 ± 1.94 pA_{rms}**
SNR (10 Hz, 5 mV_{rms} sine)	32.4 ± 3.77 dB
SNR (100 Hz, 5 mV_{rms} sine)	42.3 ± 3.4 dB
SNR (1,000 Hz, 5 mV_{rms} sine)	58.6 ± 3.92 dB

Supplementary Table 2. Noise and SNR for trans-impedance amplifier circuit. The noise and SNR values of the trans-impedance amplifier combined with 1-transistor active electrode arrays for different frequencies of interest.

Supplementary Video 1. Device insertion process. The device was inserted with a flow speed of 3 mL/min for the syringe pump and an upward movement of 20 mm/min for the motorized stand.
